# Sub-micrometre accurate free-form optics by three-dimensional printing on single-mode fibres

**DOI:** 10.1038/ncomms11763

**Published:** 2016-06-24

**Authors:** Timo Gissibl, Simon Thiele, Alois Herkommer, Harald Giessen

**Affiliations:** 1 4th Physics Institute and Research Center SCoPE, University of Stuttgart, Pfaffenwaldring 57, 70569 Stuttgart, Germany; 2 Institute for Applied Optics (ITO) and Research Center SCoPE, University of Stuttgart, Pfaffenwaldring 9, 70569 Stuttgart, Germany

## Abstract

Micro-optics are widely used in numerous applications, such as beam shaping, collimation, focusing and imaging. We use femtosecond 3D printing to manufacture free-form micro-optical elements. Our method gives sub-micrometre accuracy so that direct manufacturing even on single-mode fibres is possible. We demonstrate the potential of our method by writing different collimation optics, toric lenses, free-form surfaces with polynomials of up to 10th order for intensity beam shaping, as well as chiral photonic crystals for circular polarization filtering, all aligned onto the core of the single-mode fibres. We determine the accuracy of our optics by analysing the output patterns as well as interferometrically characterizing the surfaces. We find excellent agreement with numerical calculations. 3D printing of microoptics can achieve sufficient performance that will allow for rapid prototyping and production of beam-shaping and imaging devices.

## Abstract (web summary)

Combining freeform optics with micro- and nano-optics can permit wavefront shaping, phase engineering, **k**-space and polarization control. Here, Gissibl *et al*. use femtosecond 3D printing to manufacture free-form optical elements, giving sub-micrometre accuracy so that direct manufacturing on single-mode fibres is possible.

## Graphical abstract

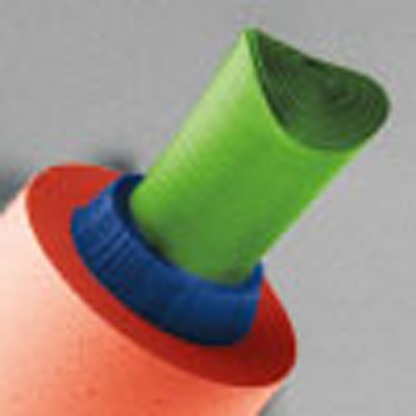

Additive manufacturing is one of the most important trends in engineering and production and will have a major impact in the twenty-first century. Direct laser writing and, in particular, multiphoton manufacturing with femtosecond laser pulses has taken additive manufacturing all the way down to the 100-nm length scale and even below^1^,^2^,^3^. Taking additive manufacturing, also known as ‘3D printing', into the optical realm would allow for simple printing of volumetric optical elements as designed. This would open the door for one of the most important topics in optical manufacturing over the last few years, namely free-form optics^4^,^5^,^6^, to enter the world of micro- and nanooptics^7^,^8^. Conventionally, free-form optics are generated by using multi-axial lathes, magnetorheological finishing, moulding and ion beam finishing techniques. Free-form optics are commonly considered as the future of modern optics, combining optical performance with systems integration^9^,^10^. A plethora of applications, such as imaging, illumination, beam shaping^11^, and particularly on the microscale, microscopy, endoscopy and inspection, would consequently be enabled. Combining free-form optics with micro- and nanooptics by combining refraction and diffraction will further permit wavefront shaping, phase engineering, **k**-space and polarization control. This is especially the case for the combination of refraction and diffraction, bringing together three-dimensional printed free-form optical surfaces with diffractive optical elements and dielectric or even plasmonic metasurfaces. Furthermore, surface structuring to achieve antireflection behaviour through graded refractive indices or deliberate scattering behaviour to diffuse beams are possible. With active control using functional materials^12^,^13^ or microfluidics^14^, very flexible zoom lenses on the micrometre scale for endoscopy inside a syringe needle would thus become possible.

In this paper, we demonstrate the combination of free-form optics with three-dimensional femtosecond printing^15^,^16^ and realize a variety of optical elements on the tips of single-mode fibres. We quantify and analyse the optical performance and demonstrate that three-dimensional printing is a viable method to generate compact and integrated optical elements previously unthinkable. Particularly, the three-dimensional aspect allows for designs that include air, other materials with specific functionalities, as well as stacked and compound designs. The optic is centred on the core of the fibre, and the lens thickness is such that on the one hand sufficient collimation power is achieved and on the other hand diffraction effects do not hamper the refractive performance too much, as the beam is allowed to propagate and expand its diameter after exiting the 4.4 μm diameter fibre core.

For the combination of micro-optical elements with fibre optics, different approaches have recently been proposed using fabrication techniques such as focused ion-beam milling^17^,^18^, interference lithography^19^,^20^,^21^, nano-imprint technology^22^,^23^, photolithography^24^, polishing techniques^25^, through-fibre patterning^26^,^27^, ultraviolet curing of surface-tension-shaped droplets in combination with coreless silica fibres^28^,^29^, inject printing of photocurable polymers^30^,^31^,^32^, laser micromachining^33^,^34^ and two-photon polymerization^35^,^36^,^37^,^38^,^39^,^40^,^41^,^42^,^43^ in order to fabricate dielectric elements directly onto the end facet of an optical fibre. However, they suffer from drawbacks such as low resolution, small fabrication volumes, the difficulty to achieve necessary large optical heights because of the required glass slides or complex fabrication setups. The additional restrictions that are required by designs of optical elements result in the fact that, to our knowledge, until now no combination of the specific design, fabrication and verification of sub-micrometre accurate free-form optics has been reported. Also, free-form optics on single-mode fibres with specific design targets cannot be fabricated by most of the mentioned methods.

To validate the complete value chain from design, manufacturing and performance assessment, dip-in laser lithography with different photoresists in a multiple step process is used^44^,^45^,^46^. Using this technique, we demonstrate different applications of sub-micrometre optical components for beam collimation, generation or correction of astigmatism, beam shaping and polarization control. This allows, for example, the specific shaping of the intensity distribution directly at the output of an optical fibre by a combination of diffractive and refractive elements.

## Results

### Complex beam shaping optics

**Figure 1:**
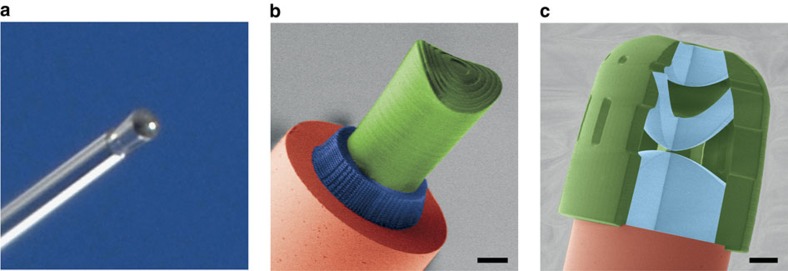
Polarization controlling, diffractive, refractive and imaging optical elements directly attached to the end facet of an optical fibre. (**a**) Macro photograph of a lens that collimates a Gaussian beam emerging from a single-mode fibre. (**b**) Coloured scanning electron microscope image of a model device manufactured by three-dimensional direct laser writing. On top of the end facet of an optical fibre (red), a chiral photonic crystal structure (blue) for polarization control and a free-form lens with Fresnel zone plate (green) for beam shaping are placed. The optical components are fabricated by two different photoresists. (**c**) Coloured scanning electron microscope image of a model device that consists of three free-form lenses (blue) and could be used on a fibre. Scale bars, 25 μm.

Figure 1 shows 3D printed optical elements where in the first case (Fig. 1a) a collimation lens has been printed onto a single-mode fibre. In addition, the photo depicts that the lens appears as transparent and glassy as the fibre. The second image (Fig. 1b) shows a free-form optical surface with a saddle-type shape aligned on a single-mode fibre. Below is a chiral three-dimensional photonic crystal structure for polarization filtering, and on top a diffractive polymer structure for beam shaping is imprinted. The three-dimensional aspect of the helical photonic bandgap material plays a crucial role in the working principle of the circular polarization filter, as will be discussed further below^47^,^48^,^49^,^50^,^51^. We demonstrate with this image the powerful diversity of capabilities when using 3D femtosecond printing in the microoptical realm. The third image (Fig. 1c) displays the possibility to include air into the design process in order to manufacture compound lenses with sophisticated aberration correction capabilities. This is something that would be completely beyond the abilities of other fabrication methods such as inkjet-type 3D printing, as used by companies such as Luxexcel^52^.

### Fabrication

Every sub-micrometre optical element present in this paper consists of commercially available photoresists that exhibit high optical quality. To fabricate our direct laser written micro-optical elements, we use a dip-in approach^44^,^45^,^46^ where the photosensitive medium is directly placed onto the objective lens. The photoresist acts simultaneously as immersion medium, and the hosting substrate can be directly dipped into the photosensitive immersion material, which is necessary for high-numerical aperture (NA) objectives. As this lithography method is not limited to small optical element volumes and limited fabrication height caused by the working distance limitations of the objective lens, our technique is well suited for such an application. The surface shapes of our free-form optics are not limited in form and complexity, as long as the feature sizes of the surface are larger than the smallest volume element, which can be exposed by two-photon absorption and as long as they are connected. After exposure, the developer has to infiltrate all parts of the structure, which need to be removed. Therefore, complex structures that contain cavities have to be properly designed. As our structures are manufactured in a layer-by-layer manner with a certain layer distance, the roughness of the surface is mainly determined by layer distance as well as proximity effects of the photoresist. In addition, this method is not limited to a specific substrate, and no complex holding and container system for the fibre and/or the photoresist is required^38^. Owing to the use of a high-NA immersion objective, feature sizes below 200 nm can easily be achieved (Fig. 5). Hence, the direct fabrication of up to cubic millimetre free-form optics with a sub-micrometre resolution on nearly any kind of hosting element or substrate can be realized.

Backside illumination of the fibre is used to accurately centre the fibre core with respect to the writing beam by observing the end facet of the fibre with a CCD (charge-coupled device) camera. This method can also be used for hybrid systems consisting of two different photoresists (Fig. 1b)^53^. In a two-step process, we first fabricate a chiral photonic crystal structure as circular polarization filter on the end facet of an optical fibre using IP-Dip (Nanoscribe GmbH), and in a second step, we use the photoresist IP-S (Nanoscribe GmbH) to create the saddle-shaped free-form lens with a Fresnel zone plate on top (Fig. 1b). The different photoresists are chosen according to their writing characteristics. Namely, we use a photoresist with a high resolution for the lower part, whereas the upper part is fabricated by a photoresist with lower resolution, such that smoother surfaces can be achieved (Fig. 1b).

### Spherical lenses for collimation

**Figure 2:**
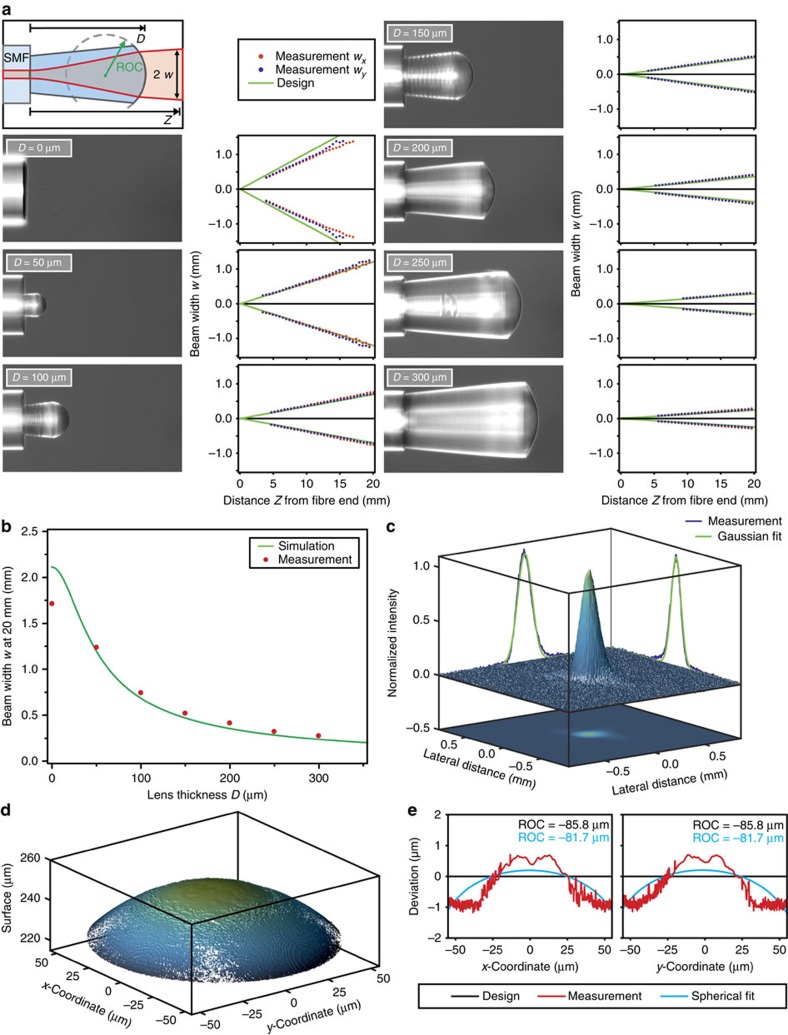
Characterization of a spherical lens for the collimation of a Gaussian beam. (**a**) Optical microscope images of different spherical lenses with different thicknesses *D* ranging from 0 to 300 μm and the corresponding propagation measurements and simulations of the beam width *w* (radius of intensity at 1/*e*^2^). The radius of curvatures are −25.6, −38.1, −53.5, −69.5, −85.8 and −102.1 μm for lens thicknesses *D* of 50, 100, 150, 200, 250 and 300 μm, respectively. (**b**) Beam width for each lens taken at a distance of 20 mm after the fibre end facet and the comparison with the simulation obtained by Gaussian beam propagation with an ABCD matrix formalism. (**c**) Intensity distribution of the beam profile at a distance of 10 mm measured from the optical fibre using a lens thickness *D* of 300 μm. (**d**) Measurement of the topography using optical interferometry of a spherical lens with a lens thickness *D* of 250 μm and a designed radius of curvature of −85.77 μm. (**e**) Deviation of the measured surface topography, which is well below 1 μm.

As a first application, we demonstrate in Fig. 2 the collimation of a Gaussian beam emerging from a single-mode optical fibre (SM 780HP, NA=0.13). Our lenses are directly fabricated on the end facet of the fibres. Spherical lenses with different radii of curvatures (ROCs) and thicknesses *D* are produced. The radii have been calculated using an ABCD matrix formalism, minimizing the beam width *w* at a distance *Z* of 20 mm after the fibre end. To achieve a collimated laser beam with minimum beam width, a certain lens thickness is required typically in the ⩾100 μm range. Otherwise, the lateral extent of the Gaussian beam is not large enough to obtain good collimation of the beam by a single refractive element^38^,^40^.

Figure 2a depicts the schematic shape and the propagation beam waist, as well as the initial fibre and six different lenses with thicknesses varying from 50 to 300 μm and ROCs from −25.6 to −102.1 μm. The plots display the designed beam waist calculated using Gaussian beam propagation, and red and blue dots denote the measured beam waist radii *w*_*x*_ and *w*_*y*_ at distance *Z* from the fibre end, respectively. The agreement is excellent. The collimation improves with increasing lens thickness, which is also verified by the simulations using the ABCD matrix formalism algorithm (green line in Fig. 2b). Good agreement between measurement and simulation in Fig. 2b and therefore excellent performance of the printed lenses is as well confirmed by the Gaussian-shaped lateral beam profile measured for a 300-μm-thick lens with a ROC of −102.1 μm at a distance of 10 mm after the fibre end (Fig. 2c).

As our structures possess strong curvatures within a small area and additionally small features sizes, characterization methods of the topography are very restricted. However, optical interferometry measurements validate the high quality of the fabricated lenses, although our structures are challenging for this kind of measurement method. In Fig. 2d, the topography of a lens with a thickness *D* of 250 μm and a ROC of −85.8 μm is shown. To manufacture a homogenous refractive index over the complete optical element, each lens is fabricated with a constant distance between the writing layers of 200 nm. The layer-by-layer fabrication process is not visible as the photoresist forms a smooth surface which makes this fabrication method excellent suited for this kind of optical elements. To accelerate fabrication, we used 800 nm writing distance and higher writing power in the lower part of the lenses. The deformation during the exposure process and the development causes small deviations between the designed and the measured surface (Fig. 2e), which are below 1 μm. This implies a maximal surface tolerance of roughly one wavelength for the designed wavelength of 800 nm. Atomic force microscopy measurements result in 43 nm root mean square (RMS) surface roughness for lenses fabricated with a layer-by-layer distance of 400 nm. This is as well confirmed by optical white light interferometry measurements. Flat films even exhibits a surface roughness of below 10 nm RMS. According to our experience, the shrinkage or deformation of such ‘bulky' objects consisting of layer-by-layer written photoresists is not uniform, as it depends on too many parameters, such as the photosensitive material, the writing parameters and the shape itself. Therefore, a prediction of the deformation is still difficult to make, especially for complex surfaces. However, the smooth surfaces and the high quality of the photoresists make it possible to reach total transmittances of up to nearly 70% for fibre pieces of up to 20 cm at a wavelength of 808 nm including the input and output coupling losses.

### Toric lenses

**Figure 3:**
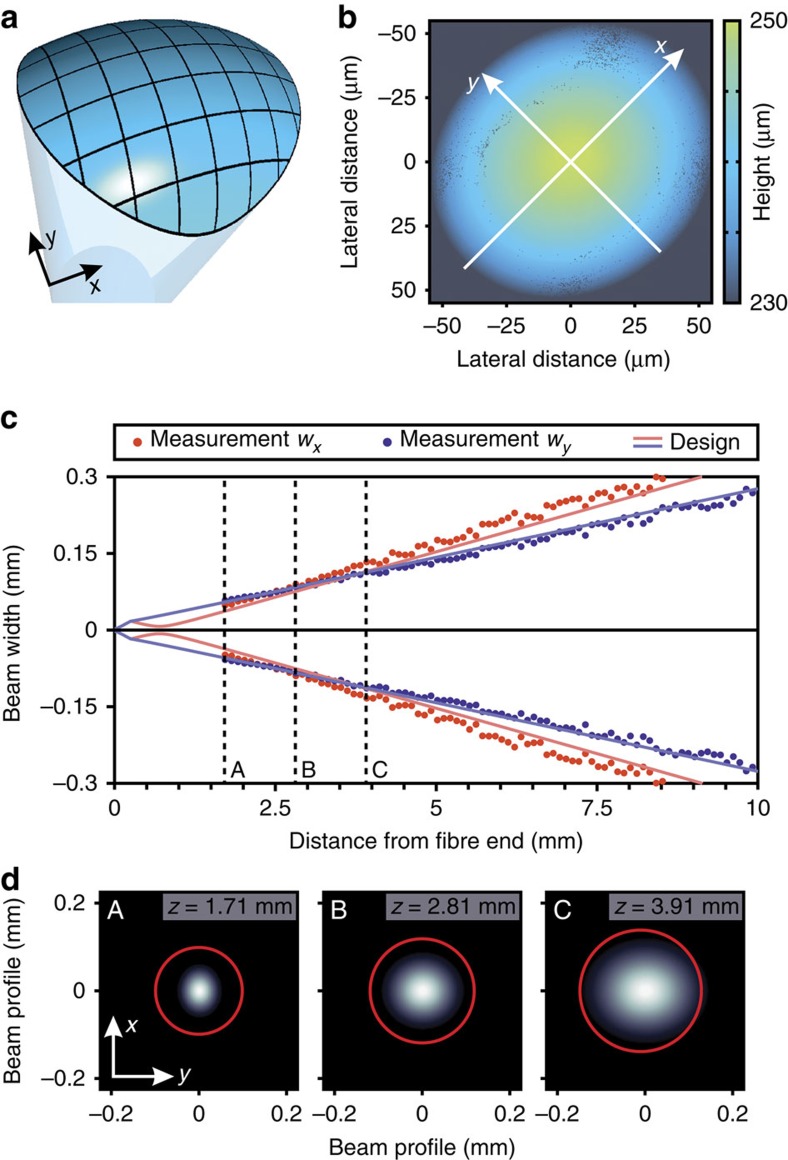
Toric lens for the generation or the correction of astigmatism. (**a**) Illustration of a toric lens that exhibits two different radii of curvature along the *x*- and the *y*-coordinate. (**b**) Measurement of the topography using optical white light interferometry of a toric lens with radii of curvature of −95.8 and −75.8 μm. (**c**) Measurement and simulation of the beam width *w* (radius of intensity at 1/*e*^2^) along the *x*- and *y*-coordinate of a toric lens with radii of curvature −65.8 and −110.8 μm, respectively. (**d**) Two-dimensional Gaussian intensity distributions fitted to the measured images of a CCD camera at different distances with respect to the optical fibre. The mode profile changes from an elliptical beam profile with the long axis in *y*-direction (A), to a spherical one (B), to an elliptical beam profile with the long axis in the *x*-direction (C).

To generate or correct astigmatism, toric lenses are usually the tool of choice. The surfaces of these lenses consist of the upper part of a torus with two different ROCs along the *x*- and the *y*-direction (Fig. 3a). Figure 3b shows the measured non-rotationally symmetric topography of such toric lens fabricated directly on a single-mode optical fibre obtained by optical white light interferometry. The two different curvatures cause different focal lengths in *x*- and *y*-direction, which results in intersecting beam waists, displayed in Fig. 3c. This behaviour is associated with the conversion of the mode profile from an elliptically shaped profile with the long axis in *y*-direction, to a spherical one at a distance of around 2.81 mm behind the fibre, to an elliptically shaped mode profile with the long axis perpendicular to the first one. This is clearly visible in Fig. 3d, which shows two-dimensional Gaussian intensity fits. For better visualization, Gaussian intensity distributions are fitted to the measured images of a CCD camera of the stigmatic beam at different distances. The red circles are guides to the eye and emphasize the change of the spatial intensity distributions.

### Free-form intensity shaping

**Figure 4:**
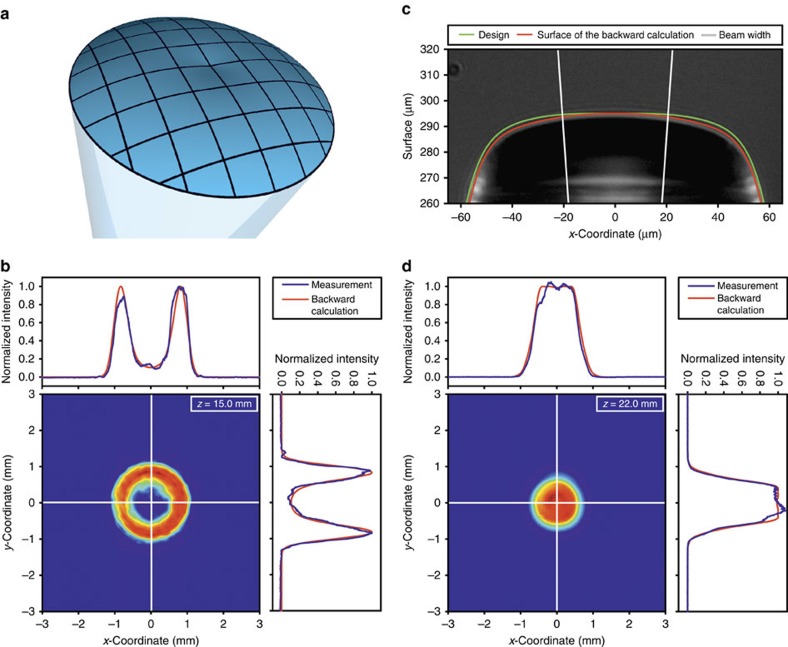
Free-form optical elements: Donut and top hat shaper. (**a**) Illustration of a donut shaping free-form lens. The lens design is calculated by an iterative optimization algorithm using Huygens–Fresnel principle. (**b**) Intensity distribution of a donut-shaped beam profile with cross sections in *x*- and *y*-direction. In addition, the backward calculation that has been determined from the measurement is shown. (**c**) Microscope image of the side view of the top hat shaping free-form optical element with indicated designed surface and the surface based on backward calculation out of the measurement results. The white vertical lines indicate the beam width. (**d**) Intensity distribution of a top hat-shaped beam profile with cross-sections in *x*- and *y*-direction. In addition, the backward calculation that has been determined from the measurement is shown.

Arbitrarily shaping the intensity distribution is one of the key goals of free-form optics. Therefore in Fig. 4 we present two different examples of sub-micrometre free-form optics directly attached to a single-mode fibre. As the calculation of free-form surfaces for optical applications is quite challenging and additionally strongly dependent on the incident illumination, different approaches to solve the redistribution of the radiation in order to create desired intensity distributions have been reported^5^,^54^,^55^. For our work, the scalar electric field at a certain distance behind the surface is calculated by numerically solving the Huygens-Fresnel diffraction integral for a given surface form^56^,^57^. With a local optimization algorithm, we iteratively improve our surface profile such that the resulting intensity distribution converges to the desired one. Using this simulation method, we design the refractive surface in order to obtain a donut shaped (Fig. 4a) and a circular top hat intensity distribution, respectively. Figure 4b,d displays the resulting two-dimensional intensity distributions after the fabrication at distances of 15 and 22 mm behind the fibre, respectively. As non-uniform deformation occurs during the fabrication and development process, the intensity distribution of the free-form surfaces are extremely sensitive to variations of the surface. They are even more responsive to deviations than the spherical or toroidal lens surfaces, as the radiation field has to be completely redistributed in some cases. Therefore, the measured intensity distribution is used in a backward calculation process where the measurement results are used in our iterative optimization algorithm in order to calculate the deformed fabricated surface. As presented in Fig. 4c, the surface of the backward calculation is in good agreement with the microscopic side view of the free-form lens, which produces a round top hat intensity distribution. The intensity distributions achieved by the backwards calculated surface (depicted in Fig. 4b,d as red curves) are in good coincidence with the measured ones as well. Fabricating a modified surface including the deviation obtained by the backward calculation can improve the surface, and therefore the agreement between measured and designed intensity distribution can be optimized. Using this procedure in an iterative algorithm will allow for the improvement of the refractive surface. More details on the deviations and measurements are included in the Supplementary Fig. 3 and Fig. 4.

### Polarization control

**Figure 5:**
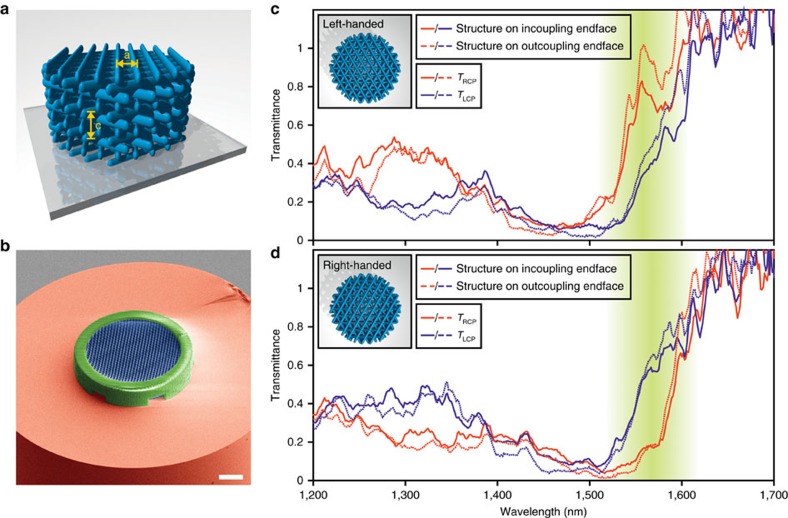
Chiral photonic crystal structure for polarization control directly on an optical fibre tip. (**a**) Illustration of the ‘twisted woodpile'. (**b**) Coloured scanning electron microscope image of the fabricated left-handed structure (blue) containing a solid ring (green) in order to increase the stability and reduce deformation of the structure. (**c**,**d**) Measured transmittance spectra for right- and left-handed circularly polarized light for the chiral photonic crystal at the in-/out-coupling end facet of the optical fibre and for the left- and right-handed photonic crystal structure, respectively. The design wavelength for the polarization filter was 1,550 nm. Scale bar, 10 μm (**b**).

Wavelength separation and polarization control are achieved by using a chiral polymer photonic crystal structure schematically shown in Fig. 5a. By using a different photoresist (IP-Dip, Nanoscribe GmbH) we obtain high resolution as visible in the colourized scanning electron microscope image (Fig. 5b) of a left-handed structure that exhibit good homogeneity. Note that the requirements of this kind of structure are completely different than for the structures presented before, however, the fabrication process is the same in both cases. The optical characteristics are displayed in Fig. 5c,d for the left-handed and right-handed structures, respectively. Around the design wavelength of 1,550 nm, the devices exhibit circular polarization selectivity with a bandwidth of roughly 50 nm. In addition, we study the influence of having the photonic crystal structure at the input and at the output coupling facet, which is negligible. As a reference, an unstructured single-mode fibre with the same length (17.5 cm) is used. Owing to slightly different fibre lengths and in-coupling efficiencies between sample and reference, the absolute values are not exactly calibrated, whereas the spectral shape corresponds to similar structures on glass^48^.

We have demonstrated various sub-micrometre optical elements, enabling intensity distribution shaping and polarization control with surface deviations between design and manufactured element of less than one wavelength. Our approach of using three-dimensional dip-in laser lithography is not limited to any kind of special optics. Furthermore, it is a fabrication platform for numerous optical elements based on diffraction, reflection and refraction in the sub-micrometre region, where among other things it is suitable for fibre technology, but similarly it is as well versatile for the combination with other substrates such as light-emitting diodes, vertical-external-cavity surface-emitting-lasers (VECSEL) and other light-emitting structures in the sub-micrometre range. Therefore, highly compact functional hybrid optical elements can be manufactured. In addition, our lenses are not limited to one refractive surface, allowing for the fabrication of optical components with two or even more compounded free-form or diffractive surfaces. Also, from the performance, we deduce that internal refractive index variations due to polymerization inhomogeneities are not detrimental to the optical performance. Our method extends additive optical manufacturing into the micro- and nanooptics realm and opens a whole new field in endoscopy and micro-imaging and -illumination.

## Methods

### Simulations

Two different simulation methods are carried out in order to describe the sub-micrometre lenses. For the simulation of the spherical and toroidal lenses in Figs 2 and 3a, Gaussian beam propagation based on ABCD matrix formalism is implemented in Matlab. For the free-form optics, as shown in Fig. 4, we use an iterative optimization algorithm in order to obtain the desired field distribution at the target plane^58^. In each iteration step, the Huygens–Fresnel diffraction integral is numerically solved in two dimensions, utilizing the rotational symmetry of the aspherical surfaces and the fields, respectively^59^. For all simulations, the refractive index of the photoresists in the exposed state is assumed to be 1.513, which is the value of OrmoComp (micro resist technology GmbH) at 800 nm wavelength. This is chosen as a first approximation and is as well confirmed by refractive index measurements carried out by measuring the critical total internal reflection angle at different wavelengths. For the beam waist of the laser beam directly after the output end facet of the single-mode fibre 780HP (Thorlabs), we use 2.45 μm, which is obtained by mode theory using the refractive indices of 1.4598 and 1.4537 for core and cladding material, respectively, and a core diameter of 4.4 μm (ref. 60). For the calculation of the designed lens surface of the top hat shaper in Fig. 4c,d, we use a different beam waist (*ω*_0_=3 μm) in order to compensate the non-uniform deformation due to the fabrication and development process.

In Fig. 2a, each ROC is determined by the minimization of the beam width *w* at a distance of 20 mm for each lens thickness. Therefore, the designed ROC are −25.6, −38.1, −53.5, −69.5, −85.8 and −102.1 μm for lens thicknesses *D* of 50, 100, 150, 200, 250 and 300 μm, respectively.

The plot of the backward calculations in Fig. 4d is carried out by using the measured intensity distribution as a target function in order to calculate and therefore control the fabricated surface by an iterative optimization algorithm. Whereas the designed surface function of the donut shaper is represented by *z*=*a*_0_+*a*_1_·*r*^1^+*a*_2_·*r*^2^+…+*a*_5_·*r*^5^ and by *z*=*a*_0_+*a*_2_·*r*^2^+*a*_4_·*r*^4^+…+*a*_10_·*r*^10^ for the top hat shaper, the backward calculated surfaces are described by a modified aspheric surface


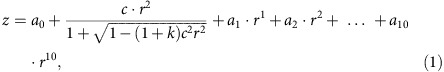


where *c* is the curvature (inverse of radius of curvature) and *k* is the conic constant. *r* is the radial distance measured from the optical axis. The parameters for the designed surface for the donut shaper are *a*_0_=0.250 mm, *a*_1_=0.109, *a*_2_=−2.258 mm^−1^, *a*_3_=−56.670 mm^−2^, *a*_4_=308.000 mm^−3^ and *a*_5_=−930.646 mm^−4^, and the backward-calculated surface is described by *a*_0_=0.250 mm, *a*_1_=8.150·10^−2^, *a*_2_=−3.430 mm^−1^, *a*_3_=−6.696 × 10^1^ mm^−2^, *a*_4_=7.639 × 10^2^ mm^−3^, *a*_5_=1.059 × 10^3^ mm^−4^, *a*_6_=1.384 × 10^−3^ mm^−5^, *a*_7_=7.024 × 10^−4^ mm^−6^, *a*_8_=−3.896 × 10^−3^ mm^−7^, *a*_9_=−9.114 × 10^−4^ mm^−5^, *a*_10_=−1.962 × 10^−4^ mm^−9^, *c*=4.164 × 10^−5^ mm^−1^ and *k*=−4.188 × 10^−4^.

*a*_0_=0.295 mm, *a*_2_=2.749 × 10^−1^ mm^−1^, *a*_4_=−3.423 × 10^3^ mm^−3^, *a*_6_=1.722 × 10^6^ mm^−5^, *a*_8_=−4.971 × 10^8^ mm^−7^ and *a*_10_=1.072 × 10^9^ mm^−9^ describe the surface of the designed top hat shaper, whereas *a*_0_=0.295 mm, *a*_1_=−5.737 × 10^−4^, *a*_2_=−2.410 mm^−1^, *a*_3_=−8.461 × 10^−2^ mm^−2^, *a*_4_=−2.010 × 10^3^ mm^−3^, *a*_5_=−1.908 × 10^−1^ mm^−4^, *a*_6_=1.557 × 10^6^ mm^−5^, *a*_7_=−4.580 mm^−6^, *a*_8_=−5.385 × 10^8^ mm^−7^, *a*_9_=9.165 × 10^−2^ mm^−5^, *a*_10_=−1.196 mm^−9^, *c*=−1.839 × 10^−3^ mm^−1^ and *k*=−1.594 × 10^−2^ describe the backward-calculated surface.

### Sample fabrication

The different optical elements are directly fabricated on the end facet of an optical single-mode fibre by three-dimensional direct laser writing. Direct laser writing uses tightly focused femtosecond laser pulses in the near infrared spectral region in order to generate two-photon absorption in an ultraviolet sensitive photoresist. As two-photon absorption is a nonlinear process, the transition rate is proportional to the square of the intensity and thus the exposure process only takes place in a small volume element around the focal spot. Therefore, arbitrary three-dimensional structures can be created by moving this small volume element through a photosensitive medium. For our purpose, a commercially available three-dimensional laser lithography system (Photonic Professional GT, Nanoscribe GmbH) is used. In contrast to many other direct laser writing applications, we use a dip-in approach^44^. To mount the fibre, we use a standard fibre holder with V-groove (Elliot Martock MDE 710) directly attached to the direct laser writing system. The fibre pieces have a length of 15–20 cm and are cleaved directly before the direct laser writing process. By backside illumination of the fibre, the core can be perfectly centred by observing the end facet of the fibre with a CCD camera and aligning the fibre core with respect to the direct laser writing beam. In this case, only a power of a few percent of the writing power is used.

In particular, for the fabrication process, a droplet of photoresist is deposited onto the objective lens and subsequently, the fibre is dipped into the photoresist. After aligning the fibre, the two-photon direct laser writing process is accomplished. To develop the structure, the unexposed photoresist has to be removed by a development bath. Using this method, the structure is perfectly aligned with respect to the core of the optical fibre. An illustration of the fabrication process can be found in Supplementary Fig. 1.

The structures shown in Figs 2 and 3 are fabricated by moving the fibre layer-by-layer with ultra-precise piezo actuators. The structures in Figs 1, 4 and 5 are manufactured by scanning the laser focus lateral by galvanometric mirrors. The vertical movement is as well carried out by piezo actuators. This method allows for fast and reproducible fabrication of sub-micrometre optical elements. In Supplementary Fig. 2, the high reproducibility of our method is demonstrated by fabricating two similar lenses with nominally identical fabrication parameters. The optical performance of the two lenses is in good agreement, which demonstrates the high reproducibility of the fabrication method.

The spherical lenses in Fig. 2 and the toric lens in Fig. 3 are fabricated using OrmoComp resist (Micro Resist Technology GmbH). In order to save writing time, every lens is separated into two parts with different layer-by-layer distances, where the upper one contains the volume with the surface that is crucial for the refraction and the lower one only represents a base. The spherical lenses in Fig. 2 and the toric lens in Fig. 3 are fabricated with a layer-by-layer distance of 800 and 200 nm for the lower and upper part, respectively. As the beam shapers in Fig. 4 are fabricated of a different material, layer-by-layer distances of 400 and 100 nm for the lower and upper part are chosen, whereas for the upper part of the top hat shaper we use a 50 nm layer-by-layer distance as this free-form optic is extremely sensitive to the surface form. For the fabrication of the free-form optics in Fig. 4, we use the photoresist IP-S from Nanoscribe GmbH. The chiral photonic crystal structures in Fig. 5 consists of IP-Dip (Nanoscribe GmbH), which is as well the photoresist of the chiral photonic crystal structure in the model device in Fig. 1b, whereas the green-coloured free-form lens with a Fresnel zone plate on top in Fig. 1b consists of OrmoComp (Micro Resist Technology GmbH). After the exposure process, a development process is carried out consisting of a 20-min bath in the respective developer (mr-dev 600 for IP-photoresists and ormoDEV for OrmcoComp, respectively, Micro Resist Technology GmbH) and a subsequent rinsing bath in isopropanol. Owing to the small sizes drying is not required. Furthermore, the used photoresists do not need any baking or other treatments before or after the exposure process. For the manufacturing of the free-form optics in Fig. 4 with heights of 250–300 μm and 120 μm the fabrication time usually is well below 2 h.

Single-mode optical fibres 780HP (Thorlabs) are used, except for the chiral photonic crystal structures, where the single-mode fibre SMF 28 (Newport) is used to support the structures.

The chiral photonic crystal structure in Fig. 5 is fabricated to have the polarization stop band at the telecommunications wavelength at around 1.55 μm. Therefore, we use a rotation angle of 120° between neighbouring layers, a rod-by-rod distance *a* of 1.25 μm, and a layer-by-layer distance of 0.44 μm, which corresponds to a lattice constant *c* of 1.32 μm. Every fourth layer is identically oriented. To overcome the ellipticity of one single rod, every rod consists of two similar written lines spaced by a distance of 100 nm. This results in a rod thickness of around 240 nm.

### Measurement of the beam width

To measure the intensity distribution of the beam profile at different distances light from a single-mode fibre-coupled laser diode with a wavelength of 808 nm (LP808-SF30, Thorlabs) is coupled into the unstructured end facet of the fibre. At several distances, pictures of the mode are taken using a CCD camera (GC 2450C, Allied Vision Technologies). The camera is moved by a linear translation stage (PI miCos GmbH). The beam width is taken at the radius where the intensity has decreased to 1/*e*^2^. In Fig. 2b, the average value of the beam width *w* in *x*- and *y*-direction at a distance of 20 mm is taken. Therefore, in some cases, linear interpolation is necessary.

### Measurement of the surface topography

The topographies of the spherical and toroidal lenses in Figs 3 and 4 are measured by using a white light interferometer system (ZygoLOT GmbH).

### Measurement of the polarization behaviour

For the polarization control measurement, the incident light from a white light source (Energetiq EQ-99 LDLS) is circularly polarized by a Glan-Thompson polarizing prism (Bernhard Halle) in combination with a broadband achromatic quarter wave plate (Bernhard Halle). A × 10 and a × 6.3 objective are used for the input and the output coupling, respectively. We investigated the influence of both situations the chiral photonic crystal structure at the input and at the output coupling end facet of the single-mode fibre, as well. The transmittance spectra are measured by using an optical spectrum analyser (Ando AQ6315E) with a sensitive region ranging from 350 to 1,750 nm.

## Additional information

**How to cite this article:** Gissibl, T. *et al*. Sub-micrometre accurate free-form optics by three-dimensional printing on single-mode fibres. *Nat. Commun.* 7:11763 doi: 10.1038/ncomms11763 (2016).

## Supplementary Material

Supplementary Information Supplementary Figures 1-4
