# Supplementary material for: Sub-micrometre accurate free-form optics by three-dimensional printing on single-mode fibres
Source: Nat Commun. 2016 Jun 24;7:11763. doi: 10.1038/ncomms11763 (PMC4931017; doi:10.1038/ncomms11763)
Supplement: Supplementary Information — Supplementary Figures 1-4 [file ncomms11763-s1.pdf]

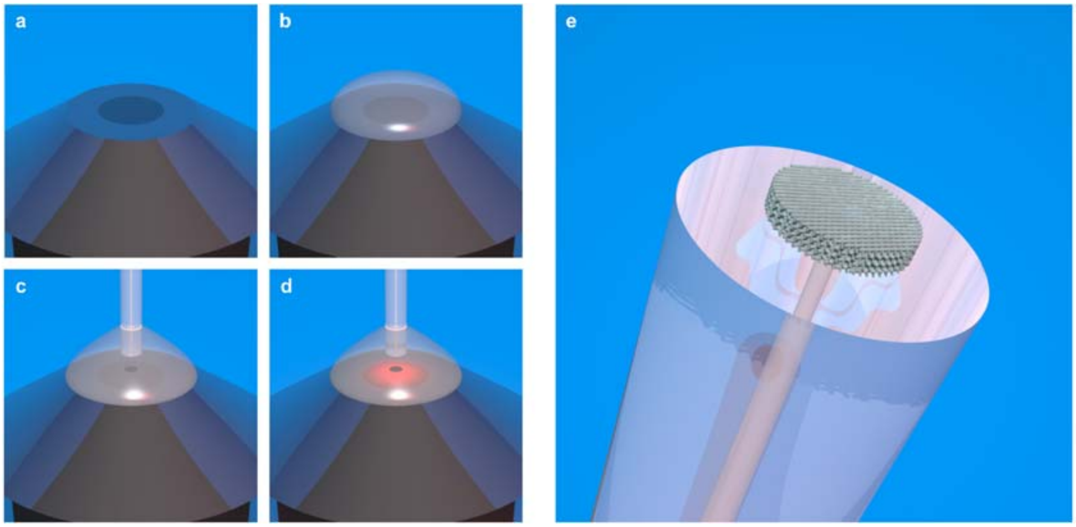

Supplementary Figure 1| Illustration of the fabrication on optical fibres using femtosecond direct laser writing. On the objective lens **(a)** a droplet of photoresist is deposited **(b)**. Subsequently, the fibre is dipped into the photosensitive medium **(c)** and accurately aligned by observing the end facet of the fibre which is illuminated from the other side with a CCD camera. In a subsequent step the structure is exposed by two-photon absorption **(d)**. After the development process the direct laser written structure is perfectly aligned with respect to the core of the optical fibre **(e)**.

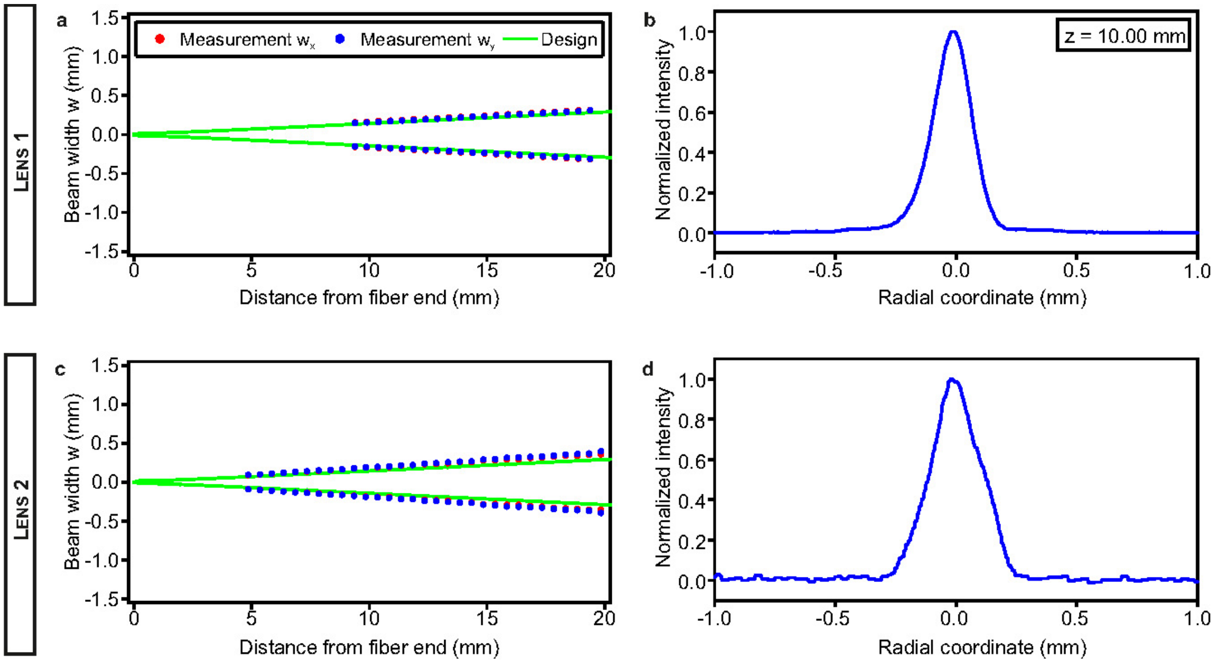

Supplementary Figure 2| Optical characterization of two similar lenses with nominal same fabrication parameters. a, Propagation measurements and simulations of the beam width w (radius of intensity at $1/e^2$) for a 250 µm thick spherical lens with a radius of curvature of -85.8 µm**. b,** Intensity profile in a distance of 10 mm behind the fibre. **c,** Propagation measurements and simulations of the beam width w (radius of intensity at $1/e^2$) for a 250 µm thick spherical lens with a radius of curvature of -85.8 µm**. d,** Intensity profile at a distance of 10 mm behind the fibre. The measured performance of the two nominal identical lenses is in good agreement, which proofs the high reproducibility of the fabrication method.

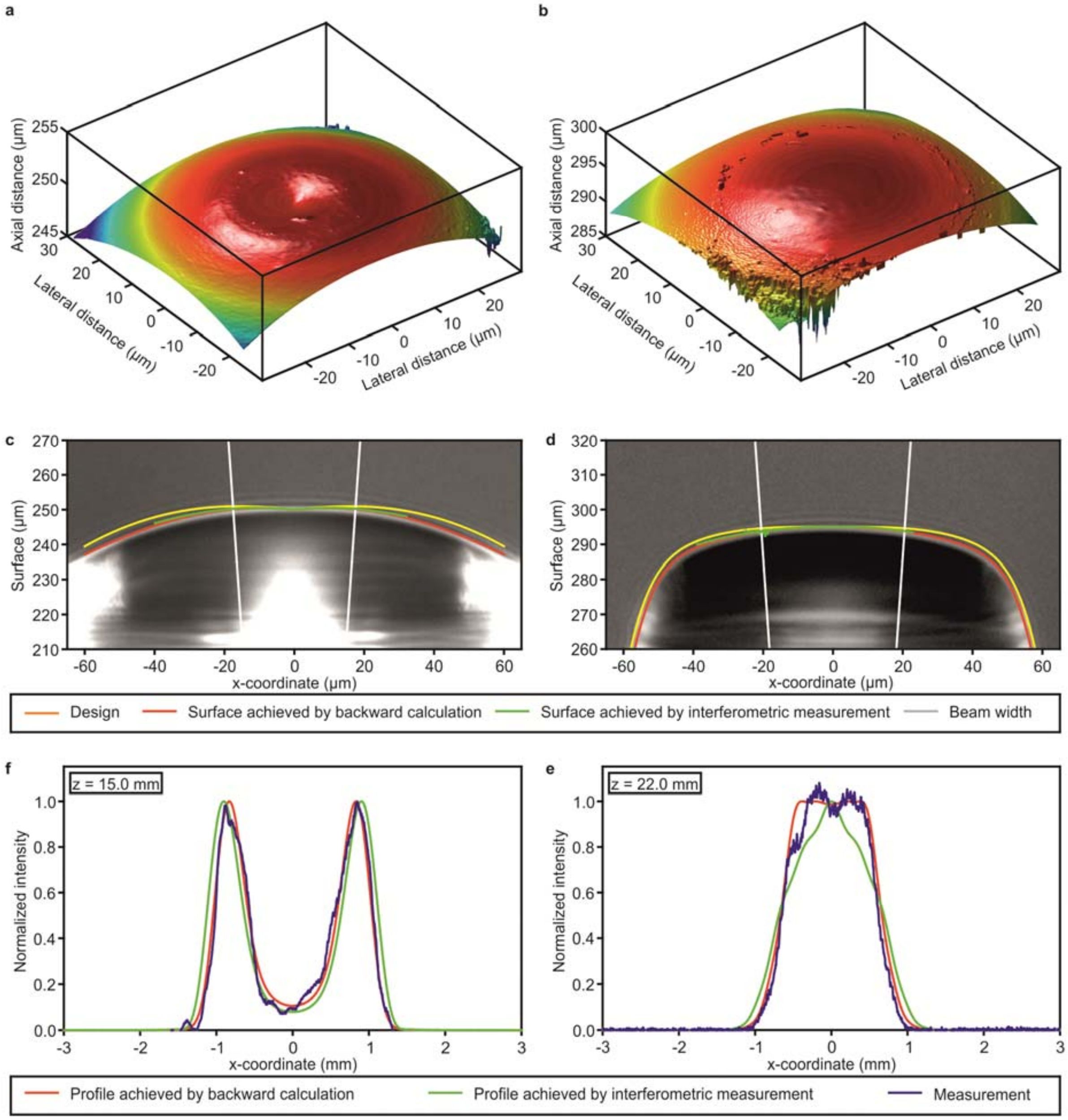

Supplementary Figure 3| Optical Linnik interferometry measurements for controlling the fabrication quality of the donut and the top hat shaping submicrometre free-form optics. a,b, Phase measurements of the topography of the donut and the top hat shaping free-form lenses, respectively. **c,d,** Comparison between the designed surface, the surfaces achieved by backward calculation, and the interferometry measurements, respectively. **f,e**, Cross sections of the measured intensity distribution of the donut shaped and the top hat shaped beam profile at a distance z of 15 mm and 22 mm, respectively. In addition, the backward calculated intensity distribution and the profile achieved by simulating the interferometry measurement data are shown.

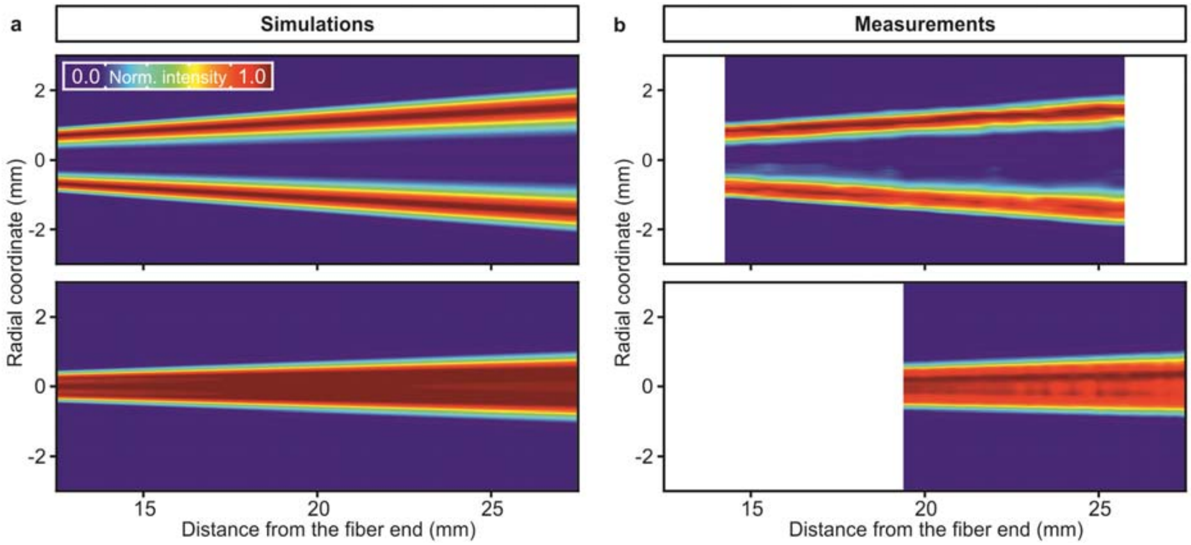

Supplementary Figure 4| Comparison between measurement and simulation of intensity distribution of the donut and the top hat shaper for different distances. a, Simulations of the intensity distribution of the donut (top) and the top hat shaper (bottom) at different distances after the fibre end. The intensity distribution in each step is obtained by numerically solving the Huygens-Fresnel diffraction integral in two dimensions. As surface parameters the backward-calculated parameters are used. **b,** Measurement of the intensity distribution at different distances after the fibre end for the donut and the top hat shaper. At each distances pictures of the mode are taken using a CCD camera. The cross section is obtained by taking the average of the cross section in x- and y-direction.
